# Search for key genes, key signaling pathways, and immune cell infiltration in uterine fibroids by bioinformatics analysis

**DOI:** 10.1097/MD.0000000000033815

**Published:** 2023-05-17

**Authors:** Feng Li, Junqing Wang, Wenqiong Liu

**Affiliations:** a Shandong University of Traditional Chinese Medicine, Shandong, China; b The Second Affiliated Hospital of Shandong University of Traditional Chinese Medicine, Shandong, China; c Affiliated Hospital of Shandong University of Traditional Chinese Medicine, Shandong, China.

**Keywords:** bioinformatics analysis, differentially expressed genes, uterine fibroids

## Abstract

Uterine fibroids grow in the myometrium and are benign tumors. The etiology and molecular mechanism are not fully understood. Here, we hope to study the potential pathogenesis of uterine fibroids by bioinformatics. Our aim is to search for the key genes, signaling pathways and immune infiltration about the development of uterine fibroids. The GSE593 expression profile was downloaded from the Gene Expression Omnibus database, which contains 10 samples, including 5 uterine fibroids samples and 5 normal controls. Bioinformatics methods were used to find differentially expressed genes (DEGs) in tissues and further analyze the DEGs. R (version 4.2.1) software was used for Kyoto Encyclopedia of Genes and Genomes (KEGG) and Gene Ontology (GO) pathway enrichment analysis of DEGs in uterine leiomyoma tissues and normal control. STRING database was used to generate protein-protein interaction (PPI) networks of key genes. Then, CIBERSORT was used to assess the infiltration of immune cells in uterine fibroids. A total of 834 DEGs were identified, of which 465 were up-regulated and 369 were down-regulated. GO andKEGG pathway analysis showed that the DEGs were mainly concentrated in extracellular matrix and cytokine related signaling pathways. We identified 30 key genes in DEGs from the PPI network. There were some differences in infiltration immunity between the 2 tissues. This study indicated that screening key genes, signaling pathways and immune infiltration by comprehensive bioinformatics analysis is helpful to understand the molecular mechanism of uterine fibroids and provide new insights into understanding the molecular mechanism.

## 1. Introduction

Uterine fibroids are one of the common gynecological diseases, affecting more than 70% of women worldwide. Although only about 25% to 30% of women with uterine fibroids have clinical symptoms,^[1]^ it can lead to reproductive dysfunction and other gynecological diseases.^[2,3]^ Therefore, uterine fibroids cannot be ignored. The occurrence of uterine fibroids has many risk factors, including race^[4]^ and age.^[5]^ Smoking and heavy drinking are also risk factors for uterine fibroids.^[6,7]^ The more risk factors, the incidence of uterine fibroids will increase. Oral or injectable contraceptives are known to reduce the risk of uterine fibroids.^[8,9]^ Studies suggest MED12 mutation is a driving factor for uterine fibroids.^[10,11]^ However, the exact basis of MED12 mutations leading to uterine fibroids is unclear.^[12]^

Gene expression microarrays have been used in numerous studies to identify DEGs and signaling pathways related to a variety of diseases. As a large-scale and efficient biological information collection technology, microarray technology can detect sequence changes in thousands of genes and monitor changes in gene expression levels across the genome. A comprehensive analysis of the interaction between key genes and enrichment pathways will help to find the potential mechanism of uterine fibroids and provide new ideas for their prevention and treatment.

We obtained the GSE593 microarray data set from the GEO database and used R (version 4.2.1) software to identify DEGs in selected uterine fibroids and normal tissues. Subsequently, we performed gene ontology (GO) and Kyoto Encyclopedia of Genes and Genomes (KEGG) pathway analysis on DEGs. Moreover, we generated a protein-protein interaction (PPI) network to analyze the association of specific proteomes. Finally, the CIBERSORT algorithm was performed between uterine fibroids and normal tissues to analyze immune infiltration. The purpose of this study was to analyze the key genes and pathways of uterine fibroids by bioinformatics, and then explore the molecular mechanism of the occurrence and development of uterine fibroids. We expect that these studies will further advance the prevention of uterine fibroids and open up new treatment ideas.

## 2. Materials and methods

### 2.1. Acquisition of microarray data and identification of DEGs

**Figure 1.**
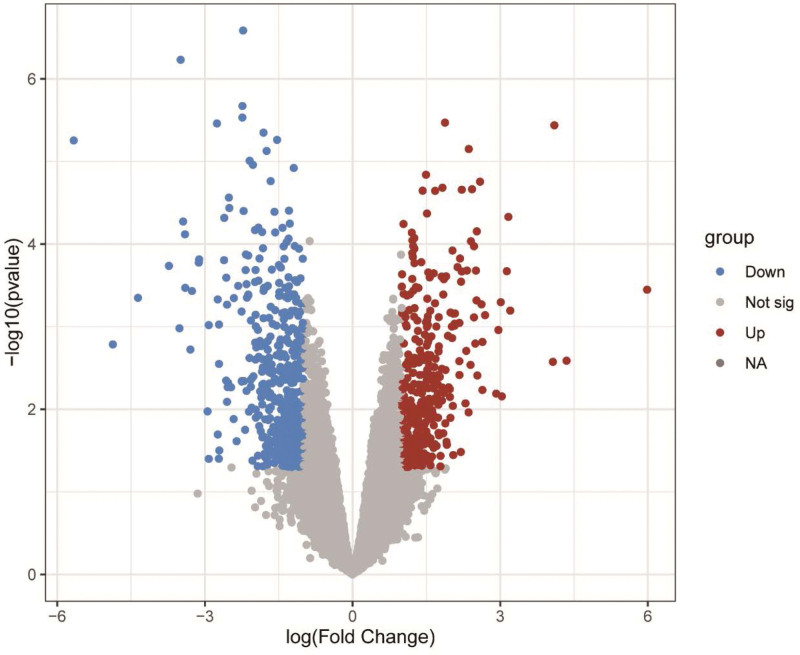
Volcano plot of the differentially expressed genes between fibroids and normal tissues.

GSE593 were downloaded from the GEO DataSets (https://www.ncbi.nlm.nih.gov/gds/), including 5 samples from normal myometrium and 5 samples from patients with uterine fibroids. The platform of GSE593 is GPL96 [HG-U133A] Affymetrix Human Genome U133A Array。 Differentially expressed genes (DEGs) were screened between normal and uterine fibroid patients by R limma package of the R software. The screening criteria for DEGs were *P* < .05 and logFC ≥ 1. And R software was used to generate a Volcano plot (Fig. 1).

### 2.2. GO and KEGG analyses

We analyzed the function and pathway enrichment of proteins encoded by key genes. GO analysis is widely used to identify cellular components, biological processes and molecular functions of genes and their products. Key genes were annotated using R (version 4.2.1). GO and KEGG analysis of DEGs was run using software packages (“BiocManager,” “clusterProfiler”). We identified DEGs that were significantly up-regulated and down-regulated. The key genes were identified from the integrated microarray uterine fibroids data, and *P* < .05 was considered statistically significant.

### 2.3. PPI network

STRING was used to explore and analyze the interactions of proteins. The network view can analyze the association of specific proteome predictions. Each network node of PPI network represents different proteins, and correlation between these nodes represents interaction of biomolecules. It can be used to identify related signaling pathways and interactions between the proteins encoded by key genes in uterine fibroids. The central node is most related to other proteins. The proteins represented by these central nodes play an important role.

*P* value of up- and down-regulated key genes not exceeding 0.05, and the key genes were submitted to the search tool (STRING: http://STRING-db.org) to retrieve the interaction genes. A hidden network disconnected node was selected in the Web page and the minimum interaction score required was set to 0.9. Cytoscape was used to calculate the Betweenness Centrality, Closeness Centrality and Degree Centrality to find key genes.

### 2.4. Immune infiltration

We used the CIBERSORT to acquire immune cells infiltration matrix. In our study, we included 22 immune cells including neutrophils, monocytes, macrophages M2, B cells memory, T cells follicular helper, plasma cells, NK cells resting, macrophages M1, mast cells activated, macrophages M0, NK cells activated, T cells CD8, mast cells resting, T cells CD4 memory resting, B cells naive, T cells CD4 naive, T cells regulatory (Tregs), T cells CD4 memory activated, dendritic cells activated, eosinophils, T cells gamma delta and dendritic cells resting. We installed the “preconditioning Core” package and used it to calculate immune cells in GSE593 expression profile. Then, “ggsci, tidyr, ggpubr” software packages were used to analyze whether there were differences between uterine fibroids and normal tissues.

## 3. Results

### 3.1. Identification of DEGs

**Figure 2.**
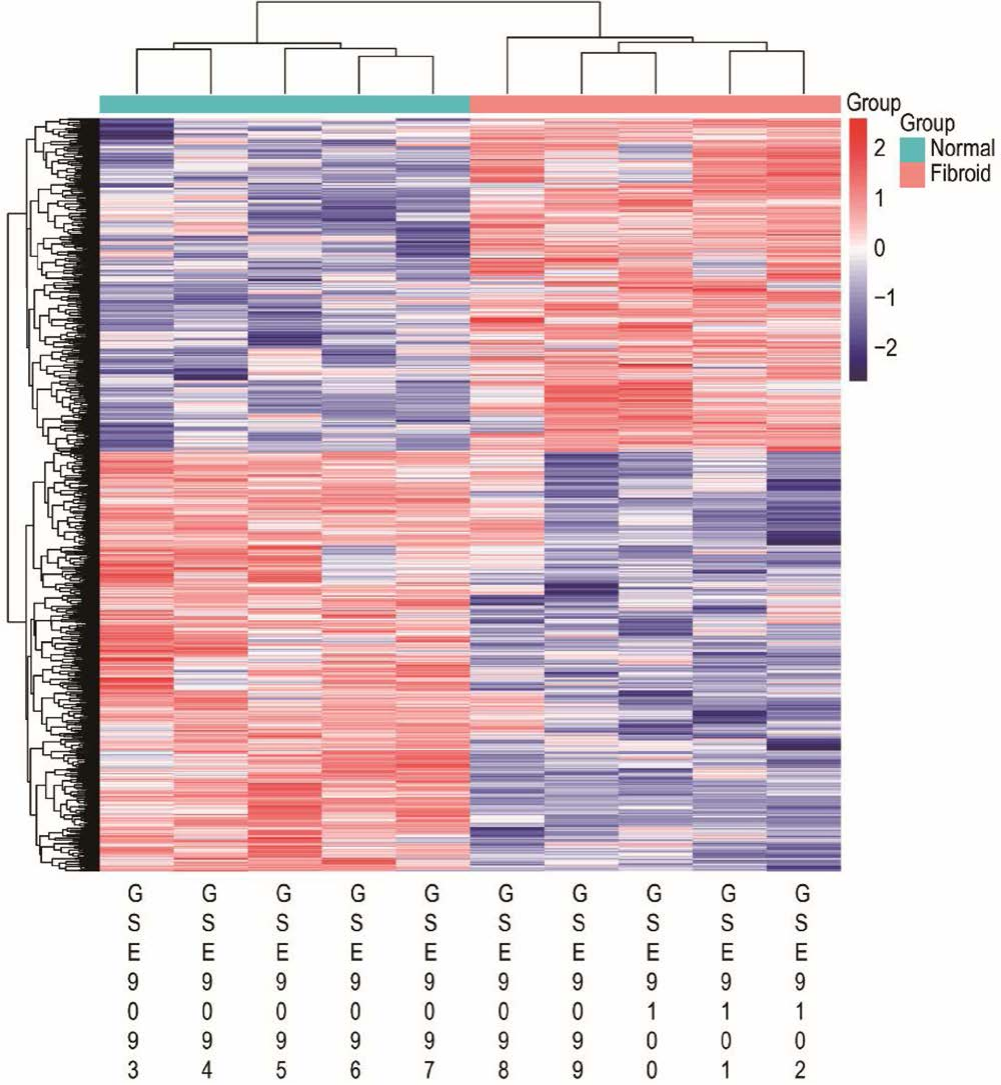
Heat map of the differentially expressed genes of GSE593. Red: up-regulation; blue: down-regulation.

The GSE593 gene expression profile consisting of 5 uterine fibroids and 5 normal samples were downloaded from the GEO DataSets. According to the standard of *P* < .05 and log2FC > 1, 834 DEGs between normal and uterine fibroids were identified by the R limma software package in R (4.2.1), including 465 down-regulated genes and 369 up-regulated genes (Fig. 1). We listed the top ten up- and down-regulated DEGs (Table 1). And the relative expression levels of DEGs were shown in heat map (Fig. 2).

**Table 1:** Top 10 of up and down-regulated differentially expressed genes in uterine fibroids.

	logFC	AveExpr	t	*P* value	adj.*P* val	B	Change
KRT19	−5.66465	4.019639	−8.3012	5.57E−06	.007038	4.317686	Down
FOSB	−4.87005	6.253501	−4.17818	.001642	.075649	−0.95662	Down
CYBRD1	−4.35933	5.754304	−4.98787	.000449	.042403	0.288441	Down
FOS	−3.73066	9.31009	−5.58137	.000184	.032258	1.136013	Down
SLC2A3	−3.51626	6.075046	−4.45295	.001047	.059077	−0.52332	Down
STEAP4	−3.49254	3.459569	−10.4915	5.88E-07	.003719	6.183862	Down
CCN5	−3.44211	5.510786	−6.46384	5.33E-05	.02108	2.290685	Down
ATF3	−3.4044	8.207581	−6.20096	7.63E-05	.022957	1.959764	Down
KLF4	−3.3991	6.011781	−5.1706	.000339	.038699	0.555472	Down
SOCS3	−3.29431	5.02703	−4.09279	.001893	.081675	−1.09332	Down
	logFC	AveExpr	t	*P* value	adj.*P* val	B	Change
GRIA2	5.983801	6.779831	5.135959	.000357	.039977	0.505262	Up
CAPN6	4.347698	7.583619	3.907059	.002585	.093671	−1.39374	Up
KIF5C	4.099792	4.125626	8.681977	3.65E-06	.006593	4.680892	Up
PART1	4.069974	5.975314	3.888788	.002667	.094773	−1.42351	Up
STMN2	3.203271	3.629988	4.760783	.00064	.049301	−0.05081	Up
PLP1	3.165657	7.494676	6.560621	4.69E-05	.019674	2.409788	Up
PCP4	3.134438	10.64076	5.480206	.000213	.033131	0.99559	Up
IL17B	3.032149	4.80149	3.330055	.006976	.14726	−2.34831	Up
ACTC1	3.012045	9.281285	4.909984	.000506	.044443	0.172998	Up
KERA	2.962597	4.917366	4.423338	.001098	.059848	−0.56951	Up

### 3.2. GO and KEGG pathway enrichment analysis

**Figure 3.**
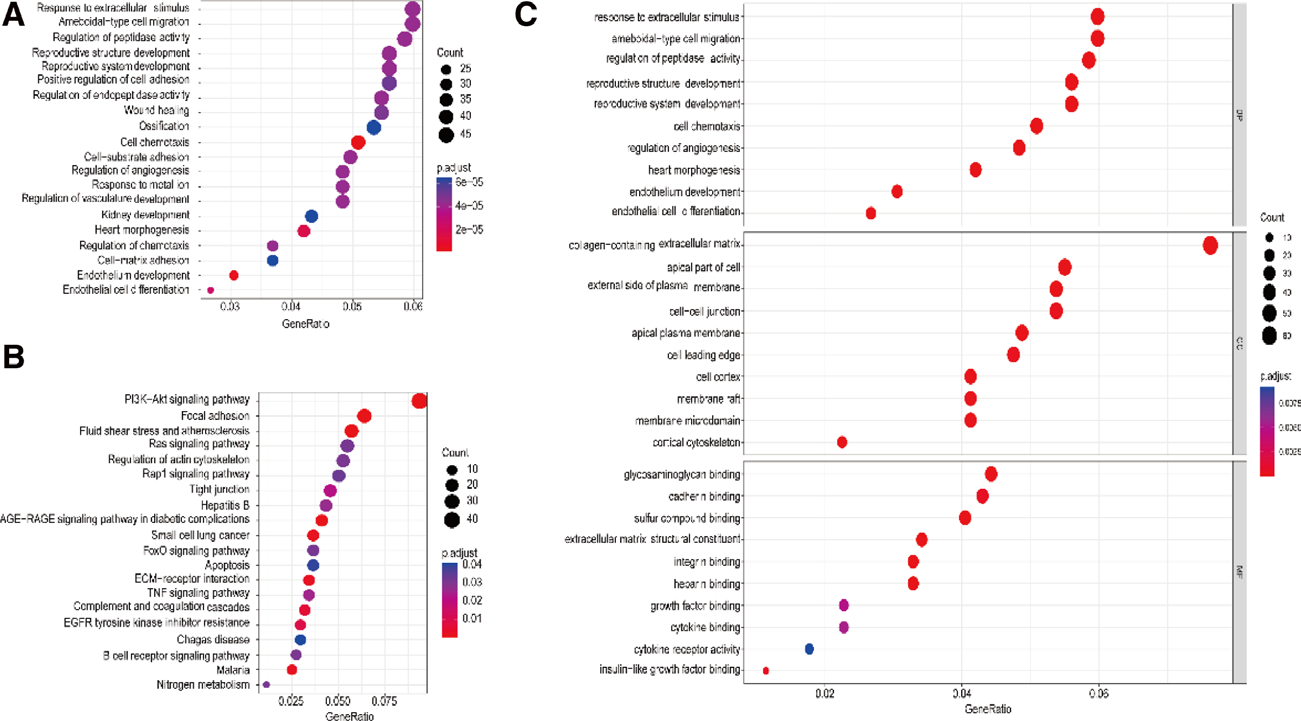
GO and KEGG pathway enrichment analysis of DEGs in GSE593. (A) GO analysis. (B) KEGG analysis. (C) Functional enrichment analysis (BP = biological processes, CC = cellular components, DEGs = differentially expressed genes, GO = Gene Ontology, KEGG = Kyoto Encyclopedia of Genes and Genomes, MF = molecular functions).

GO and KEGG pathway enrichment analysis was performed on differential genes. Table 2 and Figure 3A show the results of the GO term in uterine fibroids. Table 3 and Figure 3B show the results of KEGG enrichment analysis of DEGs in uterine fibroids. And Figure 3C shows the results of functional enrichment analysis of DEGs in uterine fibroids. According to GO analysis, the DEGs were mainly enriched in reproductive structure development, collagen-containing extracellular matrix, response to extracellular stimulus, ameboidal-type cell migration, positive regulation of cell adhesion, regulation of peptidase activity, cell growth, reproductive system development, apical part of cell and regulation of endopeptidase activity. In KEGG analysis, DEGs are mainly enriched in PI3K-Akt signaling pathway, Focal adhesion, MAPK signaling pathway, Cytokine-cytokine receptor interaction, Ras signaling pathway, Regulation of actin cytoskeleton, Rap1 signaling pathway, Fluid shear stress and atherosclerosis, cAMP signaling pathway and Tight junction.

**Table 2:** Gene ontology function enrichment analysis of 10 DEGs.

GO ID	Ontology	Description	Count	*P* value	p.adjust
GO:0062023	CC	Collagen-containing extracellular matrix	61	1.29E-17	9.61E-15
GO:0009991	BP	Response to extracellular stimulus	47	5.43E-08	4.29E-05
GO:0001667	BP	Ameboidal-type cell migration	47	5.78E-08	4.29E-05
GO:0052547	BP	Regulation of peptidase activity	46	3.28E-08	4.29E-05
GO:0016049	BP	Cell growth	46	1.92E-07	6.48E-05
GO:0048608	BP	Reproductive structure development	44	5.30E-08	4.29E-05
GO:0061458	BP	Reproductive system development	44	6.48E-08	4.29E-05
GO:0045785	BP	Positive regulation of cell adhesion	44	1.24E-07	5.18E-05
GO:0045177	CC	Apical part of cell	44	1.46E-08	5.42E-06
GO:0052548	BP	Regulation of endopeptidase activity	43	9.10E-08	4.29E-05

BP = biological processes, CC = cellular components, DEGs = differentially expressed genes, GO = Gene Ontology.

**Table 3:** KEGG pathway enrichment of 10 DEGs.

KEGG ID	Description	Count	*P* value	p.adjust
hsa04151	PI3K-Akt signaling pathway	41	2.10E-06	0.000292
hsa04510	Focal adhesion	28	2.84E-06	0.000292
hsa04010	MAPK signaling pathway	27	.004258	0.045262
hsa04060	Cytokine-cytokine receptor interaction	27	.004459	0.045776
hsa05418	Fluid shear stress and atherosclerosis	25	7.23E-08	2.23E-05
hsa04014	Ras signaling pathway	24	.001749	0.031681
hsa04810	Regulation of actin cytoskeleton	23	.001409	0.031009
hsa04015	Rap1 signaling pathway	22	.001945	0.033283
hsa04024	cAMP signaling pathway	22	.003672	0.041887
hsa04530	Tight junction	20	.000692	0.021299

DEGs = differentially expressed genes, KEGG = Kyoto Encyclopedia of Genes and Genomes.

This enriched information will help us to further understand the mechanism of the development of uterine fibroids and to study the importance of DEGs in uterine fibroids.

### 3.3. Analysis results of PPI networks

**Figure 4.**
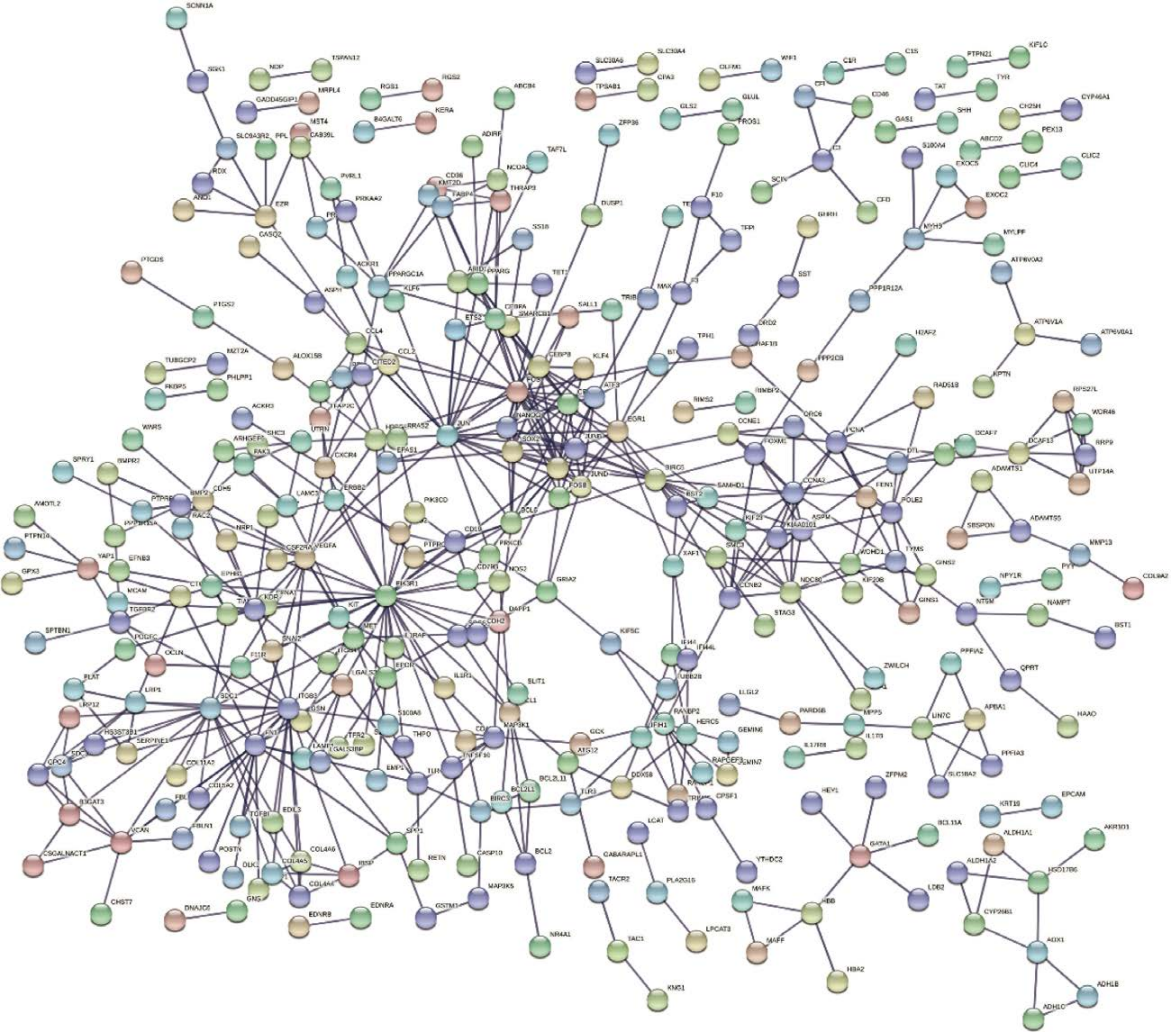
A PPI network. PPI = protein-protein interaction.

**Figure 5.**
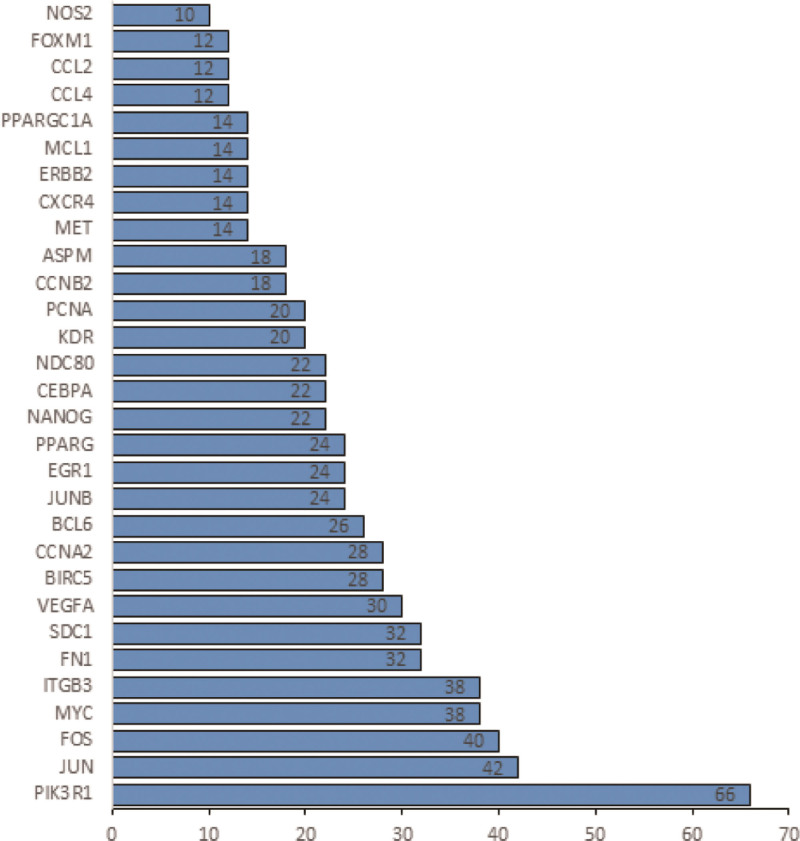
The predicted association rank of the 30 most significant genes in the PPI network. PPI = protein-protein interaction.

DEGs expression products built PPI networks through the STRING database. The minimum interaction score of 0.90 was selected and disconnected and isolated nodes were deleted. Then the network was constructed (Fig. 4). We selected top 30 of significant genes showing statistically significant interactions, which were PIK3R1, JUN, FOS, MYC, ITGB3, FN1, SDC1, VEGFA, BIRCS, CCNA2, BCL6, JUNB, EGR1, PPARG, NANOG, CEBPA, NDC80, KDR, proliferating cell nuclear antigen (PCNA), CCNB2, ASPM, MET, CXCR4, ERBB2, MCL1, PPARGC1A, CCL4, CCL2, FOXM1, NOS2 (Fig. 5). Among these genes, PIK3R1, JUN and FOX have the highest correlation.

### 3.4. Immune infiltration analyses

**Figure 6.**
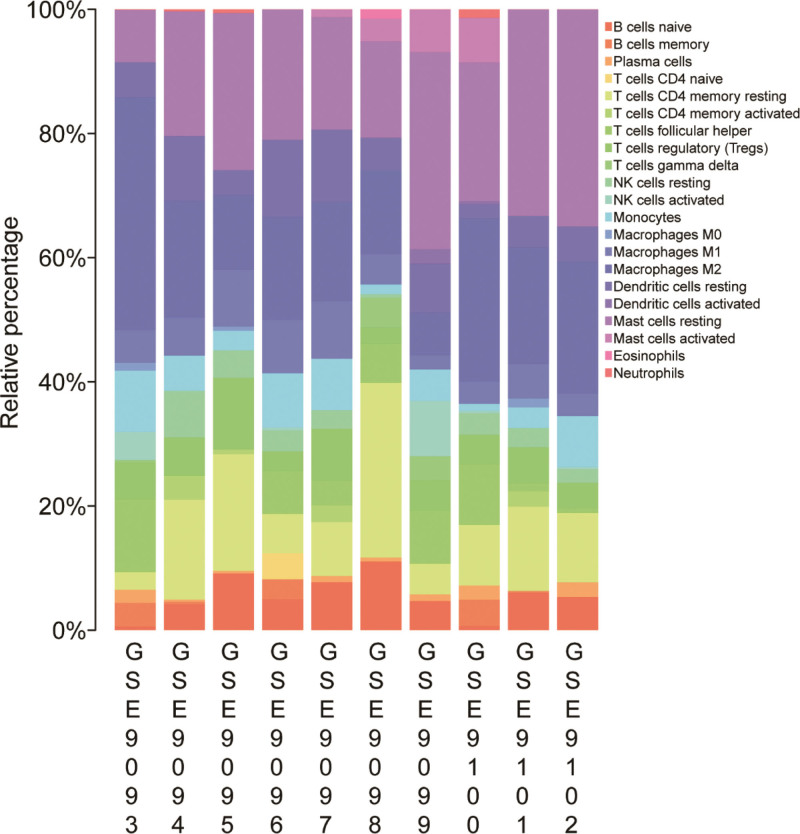
Landscape of immune cell infiltration.

**Figure 7.**
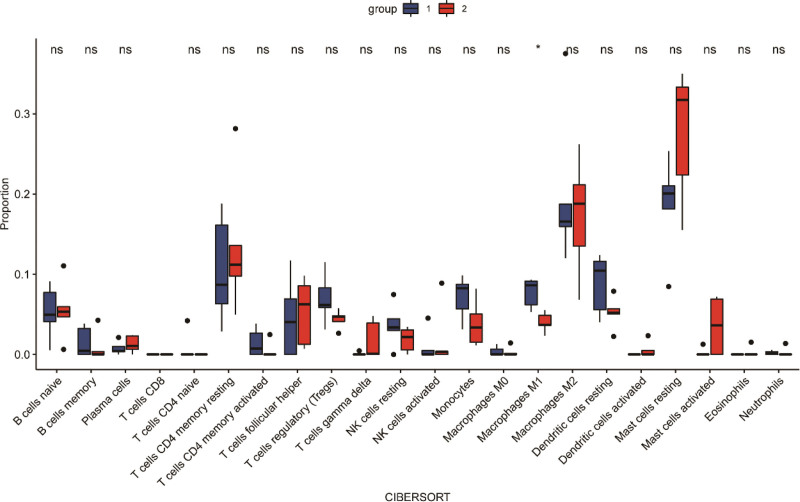
The immune cell proportions in 2 tissues. Red: fibroids tissues; blue: normal tissues.

Figure 6 shows the relative percentage of 22 immune cells. And Figure 7 shows that only the decrease of macrophage M1 in uterine fibroids was statistically significant compared with normal tissues (*P* < .05).

## 4. Discussion

Uterine fibroids are benign monoclonal tumors of the myometrium. Although benign, uterine fibroids have a significant incidence and are the main indication for hysterectomy.^[2]^ Uterine fibroids occur in women of childbearing age, which is one of the most common benign pelvic tumors. About 70% of women worldwide are affected by it.^[13]^ At the same time, age, race and obesity are risk factors for uterine fibroids.^[4,5,14]^ Many patients with uterine fibroids are asymptomatic or have mild symptoms, and only about 30% of women have clinical symptoms of uterine fibroids.^[1]^ Uterine fibroids are widespread and mostly have no obvious clinical symptoms but are the main source of gynecological and reproductive dysfunction.^[2,3]^ Therefore, it is very important to study and observe the mechanism and development of uterine fibroids at the molecular level. So, the application of genome technology has identified differential genes responsible for the formation of uterine fibroids.

A high frequency of mediator complex subunit 12 (MED12) mutations was observed in tumors from women of different races, suggesting that MED12 is a major common driver of uterine fibroids,^[10]^ MED12 mutations are closely related to race and occur more frequently in African women.^[15]^ And MED12 mutations are the real drivers of fibrogenesis.^[16]^ In addition, high mobility group AT-hook 2 triggers the pathogenesis of uterine leiomyoma by activating proto-oncogene pleomorphic adenoma gene 1.^[17]^ Compared with myometrium, the expression level of the high mobility group A2 was increased in uterine leiomyoma.^[18]^ Fumarate hydratase mutation found in uterine leiomyoma.^[18]^ Fumarate hydratase deletion changes expression profile in patients with uterine leiomyoma.^[19]^ In addition, COL4A5/COL4A6 deletion is a rare alternative that accounts for about 2% of uterine fibroids.^[20]^ YEATS4 and ZNHIT1 are members of SRCAP, and their germline mutations occur in patients with uterine fibroids.^[16]^

We collated gene expression profile data sets for GSE593 and analyzed them using R software (version 4.2.1). A total of 834 DEGs were identified. There were 465 genes up-regulated and 369 down-regulated. Among them, the most significantly up-regulated 20 genes were GRIA2, CAPN6, KIF5C, PART1, STMN2, PLP1, PCP4, IL17B, ACTC1, KERA, KRT17, DCX, TPH1, PPP1R1A, CCN6, COPG2IT1, RPE65, SAC3D1, DKK2, and TACR2. The 20 most significantly downregulated genes were ADH1B, GAS1, BTG2, GABPA, FKBP5, PTGS2, CCL2, ZFP36, PPL, SPTBN1, SOCS3, KLF4, ATF3, CCN5, STEAP4, SLC2A3, FOS, CYBRD1, FOSB, and KRT19. GO analysis of DEGs was performed by R (4.2.1) software. The GO analysis suggested key genes specifically involved in reproductive structure development, collagen-containing extracellular matrix, response to extracellular stimulus, ameboidal-type cell migration, regulation of peptidase activity, cell growth, reproductive system development, positive regulation of cell adhesion, apical part of cell, and regulation of endopeptidase activity. This finding suggests that extracellular matrix and cell overgrowth play an important role in the occurrence of uterine fibroids. Excessive extracellular matrix (ECM) play a role in the formation of uterine fibroids; a large number of ECM deposition is the characteristics of uterine fibroid cells. The key to fibrotic diseases such as uterine fibroids is the abnormal remodeling and massive accumulation of ECM. In uterine fibroids, ECM deposition is based on highly cross-linked interstitial collagen and abundant glycosaminoglycans.^[21]^ The formation of uterine fibroids is involved in the Wnt/β-catenin signaling pathway, and the Wnt/β-catenin signaling pathway also has a variety of physiological processes, such as tissue renewal, cell differentiation and proliferation.^[22]^ In uterine fibroids, ECM was also found to activate β-catenin signaling.^[23]^ And the use of vitamin D3 can inhibit the Wnt/β-catenin pathway and reduce the proliferation of uterine leiomyoma cell.^[24]^

KEGG pathway analysis displayed that key genes were mainly enriched in Cytokine-cytokine receptor interaction, Focal adhesion, Ras signaling pathway, MAPK signaling pathway, PI3K-Akt signaling pathway, Fluid shear stress and atherosclerosis, Regulation of actin cytoskeleton, Rap1 signaling pathway, cAMP signaling pathway and Tight junction. Many biological events interact with each other. The incidence of uterine fibroids is affected by the interaction of multiple abnormal pathways. It can be seen that many biological events interact with each other. For example, the expression of β-catenin responsive genes in uterine leiomyoma cells is achieved by estrogen signaling to activate the β-catenin pathway. This pathway induces nuclear translocation of β-catenin. Studies have shown that dietary factors cause vitamin D deficiency in mice, which induces myometrium inflammation.^[25]^ The mechanism by which vitamin D3 inhibits uterine fibroids is related to its effect on ECM deposition, pro-inflammatory pathways, and Wnt/β-catenin in uterine fibroid.^[26]^ In addition, Enhances DNA damage and inhibits repair of damaged DNA by reducing VDR in human myometrium cell.^[27]^ These studies have shown that vitamin D3 interacts with the β-catenin pathway and affects DNA damage and repair.

PPI network of proteins encoded by key genes was constructed. Then, we identified the first 30 related key genes: PIK3R1, JUN, FOS, MYC, ITGB3, FN1, SDC1, VEGFA, BIRCS, CCNA2, BCL6, JUNB, EGR1, PPARG, NANOG, CEBPA, NDC80, KDR, PCNA, CCNB2, ASPM, MET, CXCR4, ERBB2, MCL1, PPARGC1A, CCL4, CCL2, FOXM1, and NOS2. Activator protein 1 is a homodimer or heterodimer composed of FOS and JUN members. This transcription factor family is involved in some biological processes, including apoptosis, differentiation, cell proliferation and a series of fibrotic diseases.^[16]^ And studies have found that ECM deposition is also associated with Activator protein 1^[28]^ and ECM activates β-catenin signal in uterine leiomyoma.^[23]^ Vitamin D also inhibits the growth and proliferation of uterine fibroid cells by inhibiting the expression of catechol-O-methyltransferase. These pathways are achieved by down-regulating cyclin-dependent kinase 1, PCNA, and B-cell lymphoma 2.^[29]^

Formation of uterine fibroids is associated with chronic inflammatory immune system.^[30,31]^ Chronic inflammatory immune characteristics can affect the occurrence and development of uterine fibroids because it will lead to an intensified immune system response in the female uterus, which will induce cell proliferation and fibrosis.^[31]^ Infiltrating immune cells have received more and more attention, which also has special significance in patients with uterine fibroids. More CD68-positive macrophages were found in uterine fibroids and surrounding myometrium than in distant myometrium. However, no difference was observed of MCT-positive mast cells and CD45-positive leukocytes between uterine leiomyomas and normal tissues.^[32]^ The number of circulating follicular helper T cells (Tfh), regulatory T cells (Treg, CD4 +) and CD4 + CD8 + T cells in patients increased significantly, while the number of T cells (CD4-CD8-) and natural killer cells (NK) decreased.^[33]^ In our study, using the CIBERSORT analysis tool, we analyzed the content and percentage of 22 immune cells in uterine fibroids. We found that the proportion of macrophage M1 in normal synovial tissue was higher.

In the past few years, bioinformatics analysis has been used as an effective research method in medical research. The results of bioinformatics analysis are helpful to further understand the pathogenesis of uterine fibroids. However, no experimental verification is the biggest shortcoming of bioinformatics analysis, which requires further experimental research.

## 5. Conclusion

Studying the interaction between DEGs by means of bioinformatics is helpful to further examine and understand the related mechanisms of the occurrence and development of uterine fibroids. These findings are helpful for us to further deepen the mechanism of uterine fibroids. However, experimental validation of molecular biology is essential for understanding the function of the key genes associated with uterine fibroids.

## Author contributions

**Writing – original draft:** Feng Li.

**Writing – review & editing:** Junqing Wang, Wenqiong Liu.
